# Characterizing Intraruminal Differences of the pH and Fermentation in Cattle

**DOI:** 10.1111/asj.70226

**Published:** 2026-07-26

**Authors:** Elsayed Mickdam, Ezequias Castillo‐Lopez, Raul Rivera‐Chacon, Qendrim Zebeli

**Affiliations:** ^1^ Nutrition and Clinical Nutrition Department, Faculty of Veterinary Medicine South Valley University Qena Egypt; ^2^ Centre for Animal Nutrition and Welfare University of Veterinary Medicine Vienna Vienna Austria

**Keywords:** acidosis, free ruminal liquid, particle associated ruminal liquid, ruminal pH

## Abstract

This study characterized the association between the ventral and dorsal ruminal pH in cows. The experiment was conducted with six nonlactating rumen‐cannulated Holstein cows fed either a grain‐rich diet (65% concentrate in dry matter) or a forage‐only diet. The dorsal and ventral ruminal pHs were measured in each cow using two identical indwelling systems, which recorded pH every 5 min. Furthermore, the volatile fatty acid concentrations and osmolality were determined in free ruminal liquid (FRL) of the ventral rumen and dorsal mat particle‐associated ruminal liquid (PARL) and in feces. Precision and accuracy properties of dorsal ruminal pH and ventral ruminal pH as a measure of reproducibility were statistically evaluated. Data showed that the concordance correlation coefficient, representing the strength of the relationship between the ventral and dorsal ruminal pHs, was high when feeding the grain‐rich diet (0.857), but low when feeding the forage‐based diet (0.467), with accuracy of 0.917 and 0.525, and precision 0.935 and 0.890 for grain‐rich diet and forage‐based diet, respectively. The study established a relationship between ventral and dorsal ruminal pHs showing that ventral pH can serve as a proxy for dorsal pH, though this relationship demonstrated higher reproducibility only when feeding grain‐rich diets.

## 1 Introduction

The ruminal pH can influence the microbial activity and the extent of its interaction with the host animal, being a crucial factor affecting both rumen and animal health and performance (Zebeli and Metzler‐Zebeli 2012). It is, therefore, the closest indicator of rumen health, functioning, and disturbances (Enemark 2008). However, there are well known intra‐ruminal differences in the pH, in a way that the pH of the rumen mat (i.e., particle‐associated ruminal liquid; **PARL**) is usually lower than the pH in the ventral rumen sac (Tafaj et al. 2004), and the latter is lower than the pH of the reticulum (Neubauer et al. 2018). Thus, the fact that the pH is not uniformly distributed across the rumen (Duffield et al. 2004), characterizing these differences is important in predicting the risk of subacute rumen acidosis (**SARA**), and, most importantly, the consequences of SARA on the rumen fiber degradation and health. Thus, it is particularly important to predict the pH of the ruminal mat, because this would help to understand the effect of ruminal pH on fiber degradation, since the cellulolytic microbes, commonly found attached to the ruminal mat, are sensitive to low pH (Koike and Kobayashi 2009). Wireless rumen sensor technologies allow pH measurements in real time at a specific location within the rumen (Dijkstra et al. 2020), offering the opportunity to characterize ruminal pH differences (Klevenhusen et al. 2014; Neubauer et al. 2018), and commonly used on farms as well (Zschiesche et al. 2023).

During high‐grain feeding, it is expected that the fermentation is displaced from the rumen to the large intestine (Gressley et al. 2011; Plaizier et al. 2008). Yet, it is not clear if the fermentation in the ventral rumen resembles the fermentation of the dorsal rumen or how other parameters are affected by the type of diet. For example, osmolality is a crucial rumen parameter affected by the type of diet that influences the ruminal microbial population's activity, which in turn may impact the ecosystem within the rumen (Castillo‐Lopez et al. 2014; Romero‐Pérez et al. 2011). Ruminal osmolality is affected by solutes including soluble minerals, ammonia, amino acids, and soluble carbohydrates and fermentation products, with volatile fatty acids (VFA) representing 30 to 40% of the total ruminal solutes (Girard et al. 2009). Thus, high‐grain diet is expected to increase ruminal osmolality, which in turn may pull water from circulation to the gut.

The first objective of this study was to characterize ruminal pH differences using a real‐time measurement with the same type of wireless sensors in the dorsal and ventral rumen sacs in cows fed all‐forage or high grain diets. We hypothesized that ventral rumen pH will serve as a reliable predictor of dorsal rumen pH, with variations influenced by a specific diet. The second objective of this study was to evaluate changes in the fermentation within the rumen and the feces, and to compare the osmolality between different ruminal sites and evaluate whether this variable is linked with the osmolality of the saliva. The corresponding hypothesis was that salivary and ruminal osmolality would rise during high grain feeding, although these changes would differ in saliva and in various locations in the rumen.

## 2 Methods

### 2.1 Animals, Experimental Design, and Diets

All procedures involving animal handling and treatment were approved by the Institutional Ethics and Animal Welfare Committee of the University of Veterinary Medicine Vienna and the Austrian national authority according to the law for animal experiments (protocol number: BMBWF‐68.205/0003‐V/3b/2019). The relevant ethical review committee permission has been received. The experiment was part of a larger project conducted at the research dairy farm of Vetmeduni (Pottenstein, Austria) and reported earlier by Castillo‐Lopez et al. (2022). For this particular study, we used a set of 6 ruminally cannulated (100 mm i.d.; Bar Diamond, Parma, ID, USA) non‐lactating Holstein cows (999 ± 59.5 kg; mean ± SD) kept together in a loose‐housing stable with straw bedding as part of the experiment of Castillo‐Lopez et al. (2022). The cows were fed two total mixed rations (TMR), a grain‐rich and a forage‐based diet. The experiment was conducted in two sequential periods. Cows were fed a grain‐rich diet (65% concentrates and 35% forage mix on DM basis) during 10 days, followed by the forage only diet (100% forage mix on DM basis) for another 10‐day period. The forage mix in the grain‐rich diet consisted of (DM basis) 26.25% grass silage and 8.75% corn silage whereas in the forage‐based diet, the forage mix consisted of 10% grass hay, 45% corn silage, and 45% grass silage. Ingredients and nutrient composition of experimental diets are shown in Table S1. The TMRs were prepared daily before morning feeding using an automatic system (Triomatic T15, Trioliet Feeding Technology, Oldenzaal, The Netherlands). Cows had individual access to their feeders using feeding troughs equipped with electronic weighing scales and computer‐regulated access gates (Insentec B.V., Marknesse, The Netherlands) to measure individual feed intake; fresh water was offered *ad libitum*.

### 2.2 Ruminal pH Monitoring

For continuous monitoring of ruminal pH during the entire study period, each cow received two ruminal pH sensors (Dascor Inc., Escondido, CA, USA: Penner et al. 2006) at the same time. The first sensor was allowed to float in the rumen by attaching it with a fishing hook wire (30 cm in length), which was tied to the ruminal cannula lid. This allowed measurements of the pH in a radius of about 30 cm beneath the ruminal mat of the dorsal sac. The second sensor was inserted manually in the ventral rumen via rumen cannula, as recommended by the company. A weight (900 g) was attached to the pH data logger to ensure that the sensor remains in the ventral part of the rumen. Before and after measurements, the sensors were calibrated (buffers of pH 7.0 and pH 4.0) following the company's instruction protocol, and the pH drift was then corrected using the pre‐ and post‐calibration data. The sensors recorded pH every 5 min and stored the readings until data extraction and conversion from millivolts to pH, which were performed at the end of each feeding regime.

### 2.3 Saliva, Rumen Fluid and Fecal Sampling

Saliva samples were collected by aspiration from the mouth of cows at 0, 4, and 8 h after morning feeding using an automatic vacuum pump (model Kataspir 30, MEDUTEK, GmbH & Co. KG, Bremen, Germany) with the maximum suction power (−80 kPa) as described by Castillo‐Lopez et al. (2021). The pH of saliva was measured using a pH‐meter (Mettler Toledo SevenGo Portable pH Meter SG2; Mettler‐Toledo GmbH, Schwerzenbach, Switzerland), and aliquots of samples were immediately stored at −20°C for later determination of osmolality.

Ruminal fluid samples were collected from all cows during the last day of each feeding regime at 0, 4, and 8 h after morning feeding. Two different types of ruminal fluid were collected: free rumen liquid (FRL) and PARL to evaluate the fermentation profile of different locations within the rumen. First, approximately 20 mL of FRL was collected from the ventral ruminal sac using a syringe through the ruminal fistula. Afterwards, to obtain PARL, we followed the protocol suggested by Tafaj et al. (2004). In detail, approximately 200 g of solid rumen digesta from various sections of the dorsal mat were manually collected and squeezed through four layers of medical gauze to obtain the PARL. For both locations, 2 mL vials were stored at −20°C for VFA and osmolality analyses.

Fecal samples were collected after restraining the cows and cleaning the perianal area; 200 g of fresh feces were collected at 0, 4, and 8 h after morning feeding and the fecal pH was immediately measured using a pH‐meter (Mettler Toledo SevenGo Portable pH Meter SG2; Mettler‐Toledo GmbH, Schwerzenbach, Switzerland). Afterwards, fecal samples were stored in 8 mL vials at −20°C for VFA analysis and osmolality determination.

### 2.4 Determination of Volatile Fatty Acids and Osmolality

The processing of the rumen samples, as well as the analysis of VFA via gas chromatography, was conducted according to the protocols described by Castillo‐Lopez et al. (2021). Briefly, the concentration of VFA in fecal samples was determined as previously mentioned (Mickdam et al. 2016) with modification of sample preparation. The sample preparation included thawing in ice followed by addition of 1000 μL of distilled water. Afterwards, 300 μL of internal standard, followed by addition of 300 μL 25% phosphoric acid, followed by vortexing thoroughly and centrifugation at 17,000 ×*g* for 15–30 min at 4°C (centrifuge Hermle, Z 326 K). The clear supernatant was analyzed for VFA concentrations similar to FRL and PARL samples.

Salivary osmolality was determined using an osmometer (Osmomat 3000, Gonotec, Berlin, Germany), according to the procedure previously described by Castillo‐Lopez et al. (2021). Although measuring osmolality in FRL and PARL, samples were thawed on ice and centrifuged at 15,000 ×*g* for 15 min at 4°C (centrifuge Hermle, Z 326 K). The supernatants were transferred into fresh tubes, and measurements were performed with a freezing depression point osmometer (Osmomat 3000, Gonotec) using 50 μL of sample. Results were expressed in mOsm/kg.

### 2.5 Statistical Analyses

Data were analyzed by ANOVA using the MIXED procedure of SAS (version 9.4, SAS Institute, Cary, NC). Each cow (*n* = 6) was used as the experimental unit in this study. Fixed effects of the models included factors like ruminal location, diet, time relative to morning feeding, as well as diet × time interaction and the interaction between diet × location. In the statistical analysis, normal distribution was verified using Proc Univariate followed by the normal and plot options. When normality was not met, log‐transformation or square root for data with zeros values (i.e., the time with ruminal pH < 5.8 during forage feeding) was applied following evaluation with the Box–Cox transformation in the Transreg procedure. In addition, before analysis, data were checked for outliers, which were removed based on Cook's distance. Data from different days from the same cow in the same diet were processed as repeated measures with first‐order variance–covariance structure matrices. Least square means are used to provide treatment means and the largest standard error of the mean is reported. The Tukey's method was used to compare the least square means. Effects were considered as significant with *p <* 0.05 and as a trend with 0.05 *≤ p ≤* 0.10.

The Pearson correlation coefficient and Lin's concordance correlation coefficient (CCC) were used to analyze the correlations between the pH values of the dorsal and ventral rumen (NLMIXED model of SAS). In addition, calculations were also made for the location shift (*u*) and the scale shift (*μ*). In terms of scale shift, two standard deviations (SD) are expressed as a difference. *μ* expresses the difference in SD between dorsal and ventral pH; a negative *u* value indicates an overestimation of the relationship of dorsal ruminal pH, whereas a positive value indicates underestimation of the relationship. Thus, comparing the dorsal and ventral rumens' reproducibility of pH measurements is made possible by the *CCC* (Lawrence and Lin 1989). The *r* and *CCC* were interpreted according to Hinkle et al. (2003), as negligible (0.00–0.30), low (0.30–0.50), moderate (0.50–0.70), high (0.70–0.90), and substantial (0.90–1.00). The Pearson correlation coefficient was computed to analyze the correlations between saliva osmolality, FRL and PARL osmolality (NLMIXED model of SAS).

## 3 Results

### 3.1 Intra‐ruminal pH differences

Data of the ruminal pH in the different locations (dorsal and ventral) that were affected by diet are shown in Table 1. The ANOVA showed that the ruminal pH was affected by location and the diet whereas their interaction had less effect. Comparing the pH of the two locations, the data showed that daily mean ruminal pH was lower in the dorsal part compared to the ventral one (Table 1). On the other hand, the amplitude pH was higher in the dorsal rumen (*p* = 0.011). The area of ruminal pH below 5.8 was significantly greater in the dorsal rumen compared with the ventral rumen (*p* = 0.01), although the time spent below pH 5.8 did not reach statistical significance (*p* = 0.104, Table 1). In cows fed a forage‐based diet, the pH levels in the ventral part of the rumen did not reach 5.8 or 6 throughout the day (Table 1). Moreover, the daily mean pH of the dorsal part was consistently lower (6.7; *p* = 0.028) whereas the amplitude pH was higher in the dorsal part compared to the ventral part (*p* < 0.01). Also, the time during which the pH stayed below the threshold of 5.8 (281.67 min/d) or 6 (488.75 min/d) was longer in the dorsal part compared to the ventral part (140.42 and 318.33 min/d, respectively) of the rumen (*p* = 0.05; Table 1) in cows fed a grain‐based diet. No interaction between diet and location was found on ruminal pH (*p* = 0.91).

**TABLE 1:** The effect of diet (grain‐rich vs. forage‐based), location within the rumen^1^ and their interaction on ruminal pH indices in cows.

Item	Grain‐rich		Forage‐based		SEM^2^	*p*		
	Dorsal	Ventral	Dorsal	Ventral		Diet	Location	Interaction
Daily mean pH	6.22	6.32	6.70	6.81	0.051	< 0.001	0.028	0.918
Minimum pH	5.34^b^	5.65^a^	6.18^b^	6.54^a^	0.070	< 0.001	< 0.001	0.755
Maximum pH	7.10	7.01	7.07	7.08	0.044	0.669	0.357	0.250
Amplitude	1.76^a^	1.36^b^	0.88^a^	0.54^b^	0.067	< 0.001	< 0.001	0.666
Area < 5.8 (min × pH/d)	73.1^a^	18.7^b^	4.83	0	11.47	0.004	0.011	0.032
Time < 5.8 (min/d)	281.7^a^	140.4^b^	0.83	0	42.91	< 0.001	0.104	0.108
Area < 6 (min × pH/d)	149.2^a^	64.0^b^	5.79	0	20.70	< 0.001	0.031	0.058
Time < 6 (min/d)	488.7^a^	318.3^b^	9.58	0	53.69	< 0.001	0.101	0.142

*Note:* ^a,b^Means of treatments within location sharing no common superscripts are significantly different after Tukey correction (*p* < 0.05).

^1^ Location of the ruminal pH sensors (dorsal or ventral).

^2^ The largest SEM.

**FIGURE 1:**
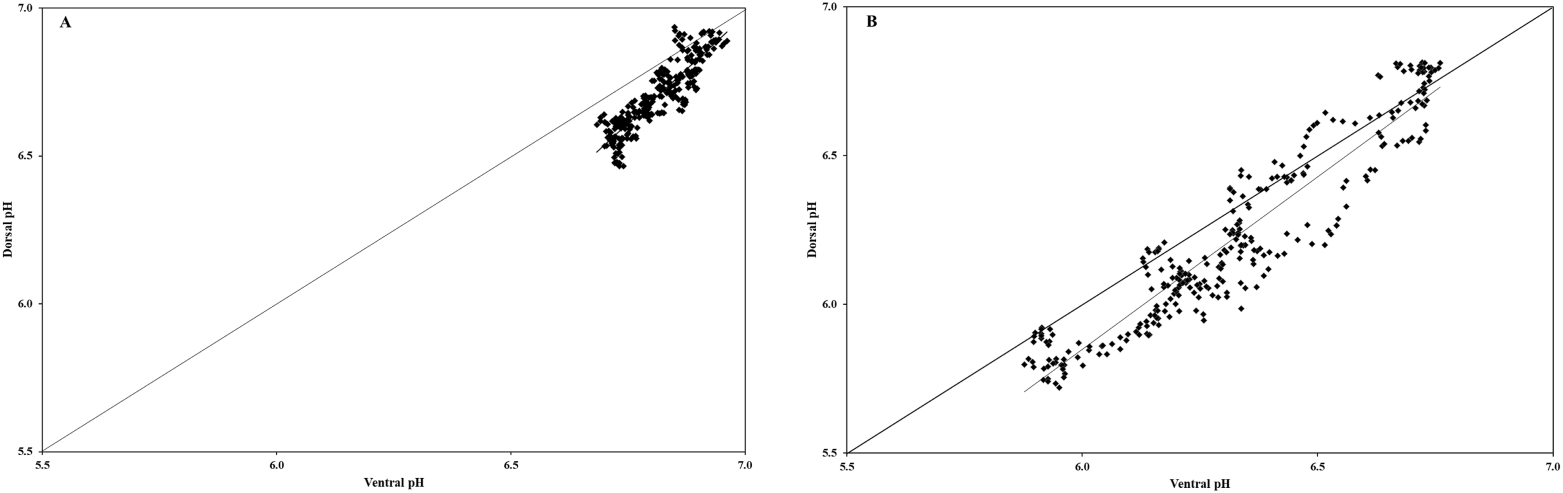
The relationships between dorsal and ventral ruminal pH measured by pH sensors in non‐lactating Holstein cows fed forage‐based diet (A) or grain‐rich diet (B). Measures of accuracy are shown in Table 2. The ideal line is also indicated (y = x).

Figure 1 shows the relationships between dorsal and ventral ruminal pH, and the measures of accuracy are shown in Table 2. Pearson correlation coefficient showed high correlations between dorsal and ventral pH values during the entire day (24 h) regardless of the diet fed (*r* = 0.935 and 0.890, for cows fed grain‐rich diet and forage‐based diet respectively, Table 2). The relationship equation from the known ventral pH readings to the corresponding dorsal pH in cows fed forage‐based diet during the whole day was: pH_dorsal_ = −3.217 + 1.445 × pH_ventral_ (Figure 1A; *p* < 0.001). The *CCC* in cows fed forage‐based diet was less precise and not accurate. On the other hand, cows fed grain‐rich diet showed substantial highly accurate and highly precise *CCC* (Table 2).

**TABLE 2:** Indices of reproducibility between pH measurements from the dorsal and ventral sac of the rumen in dairy cows fed different diets.

Item^2^	Diet^1^	
	Grain‐rich	Forage‐based
Sample size (*n*)	288	288
Pearson correlation coefficient (*r*)	0.935	0.890
Concordance correlation coefficient (*CCC*)	0.857	0.467
Accuracy	0.917	0.525
Precision	0.935	0.890
Scale shift (*μ*)	−0.156	−0.351
Location shift (*u*)	0.257	0.884

^1^ Six ruminal cannulated non‐lactating Holstein cows were fed a forage‐based diet (0% grain) or grain‐rich diet (65% grain).

^2^ The *r* and *CCC* were interpreted as negligible (0.00 to 0.30), low (0.30 to 0.50), moderate (0.50 to 0.70), high (0.70 to 0.90), and substantial (0.90 to 1.00); *μ* expresses the difference in SD between dorsal and ventral pH; negative *u* value indicates an overestimation of the relationship of dorsal ruminal pH, whereas a positive value indicates underestimation of the relationship.

### 3.2 Fermentation Profile of FRL and PARL, and the Feces

Data of fermentation profile of FRL, PARL and feces are shown in Table 3. Cows receiving a grain‐rich diet had higher concentrations of VFA in FRL compared to cows fed a forage‐based diet (*p* = 0.009; Table 3). With the exception of total VFA, isobutyrate and isovalerate (*p* = 0.001), there was no diet × time interaction in VFA profile. Molar percentages of acetate increased (+11%) at the expense of propionate (−20%) and butyrate (−26%) in cows fed a forage‐based diet (*p* = 0.001), whereas the isobutyrate and isovalerate percentages remained unaffected by diet. Acetate to propionate ratio of cows fed a grain‐rich diet was lower than that of cows fed a forage‐based diet (*p* = 0.005).

**TABLE 3:** Concentration of total and proportions of individual volatile fatty acids (VFA) in free rumen liquid (FRL), particle associated rumen liquid (PARL) and feces of cows fed grain‐rich and forage‐based dies.

Item	Diet						SEM^5^	*p*		
	Grain‐rich			Forage‐based				Diet	Time	Interaction
Time^1^	0	4	8	0	4	8				
FRL^2^										
Total VFA concentration, m*M*	78.3	113**	120**	80.6	88.5	89.7	8.19	0.009	0.001	0.010
% of total VFA										
Acetate	67.4	63.2**	61.5**	72.6	71.7	70.6	1.97	0.001	0.010	0.230
Propionate	16.5	20.4**	21.7**	14.6	15.5	16.2	1.68	0.014	0.002	0.099
Butyrate	11.5	12.3	12.9**	9.18	8.76	9.11	0.88	0.002	0.306	0.193
Valerate	1.32	1.69*	1.78**	0.96	1.19*	1.29**	0.13	0.001	0.001	0.686
Isobutyrate	1.52	1.02**	0.91**	1.18	1.17	1.12	0.09	0.942	< 0.001	0.001
Isovalerate	1.68	1.30**	1.22**	1.49	1.64	1.71**	0.16	0.140	0.178	0.001
A:P^4^	4.31	3.23**	2.94**	5.04	4.63	4.37	0.43	0.005	0.001	0.277
PARL^3^										
Total VFA concentration, m*M*	113	152**	165**	121	118	113	7.31	0.007	0.003	0.001
% of total VFA										
Acetate	65.0	62.9*	62.1*	75.1	73.1	72.2*	1.911	< 0.001	0.003	0.997
Propionate	19.4	21.2**	21.9**	14.3	15.3*	15.5*	1.78	0.010	< 0.001	0.151
Butyrate	11.1	11.9	12.1*	7.70	8.08	8.55	0.796	0.001	0.031	0.763
Valerate	1.78	1.81	1.86	0.83	1.14*	1.18**	0.131	< 0.001	0.025	0.216
Isobutyrate	1.21	0.90**	0.82**	0.90	0.97	1.07*	0.094	0.969	0.084	0.001
Isovalerate	1.55	1.29*	1.22**	1.17	1.45*	1.51**	0.154	0.901	0.993	0.001
A:P^4^	3.48	3.11	2.98*	5.33	4.79**	4.67**	0.344	0.001	< 0.001	0.701
Feces										
Total VFA concentration, m*M*	86.5	79.5	71.1*	45.2	38.9	47.2	4.98	< 0.001	0.288	0.143
% of total VFA										
Acetate	74.6	74.3	73.7	75.2	73.3*	70.2**	0.664	0.106	< 0.001	0.003
Propionate	16.0	16.1	16.8*	14.8	15.6*	16.4**	0.300	0.059	0.001	0.162
Butyrate	6.05	5.47	5.10*	4.89	5.58	6.43**	0.321	0.787	0.492	0.001
Valerate	1.38	1.68	1.53	1.57	1.52	2.35**	0.163	0.043	0.022	0.018
Isobutyrate	0.93	1.25	1.42*	1.77	2.07	2.36**	0.182	0.002	0.002	0.892
Isovalerate	1.05	1.25	1.43*	1.84	1.92	2.22*	0.262	0.056	0.012	0.841
A:P^4^	4.67	4.61	4.39*	5.10	4.70**	4.30**	0.113	0.271	< 0.001	0.023

*Note:* *,**Means of hours after morning feeding differ from 0 h, *p* < 0.05 and *p* < 0.01, respectively.

^1^ Hours after morning feeding.

^2^ Free rumen liquid.

^3^ Particle associated rumen liquid.

^4^ Acetate to propionate ratio.

^5^ The largest SEM.

Diet significantly affected VFA profile in PARL, too (Table 3). Similar to FRL, a significant interaction of diet and time was found for the concentration of total VFA, isobutyrate, and isovalerate molar percentage (*p* = 0.001). With the exception of isoacids, there were time and diet effects on other VFA profile.

Furthermore, the dietary treatment had significant influences on fecal VFA concentration (Table 3). Fecal pH was significantly influenced by dietary treatment (*p* < 0.001), with mean values decreasing from 6.69 in cows fed the forage‐based diet to 6.47 in those receiving the grain‐rich diet. There was no time effect and no diet × time interaction found for fecal pH (data is not presented). Cows receiving the grain‐rich diet showed lower fecal pH values compared with cows fed the forage‐based diet (*p* < 0.001). When cows fed the forage‐based diet, fecal pH did not differ from rumen pH at different locations (FRL and PARL) at the same time points. The concentration of total VFA in feces was higher in cows fed the grain‐rich diet (*p* < 0.001). With regard to the fecal VFA profile, acetate (*p* = 0.106) and butyrate (*p* = 0.787) proportions remained unaffected by diet whereas propionate proportion tended to increase in cows fed the grain‐rich diet (*p* = 0.059).

Correlative analysis revealed a strong negative correlation (*r* = −0.87) between total VFA concentration in FRL and feces (*p* = 0.023) whereas this correlation was not significant between PARL and feces. Moreover, the correlation between total VFA concentration in FRL and PARL was not significant.

### 3.3 Osmolality of Different Rumen Fluids and Saliva

Osmolality of the FRL, PARL and saliva are shown in Table 4. There is no time effect on osmolality of different parameters whereas the FRL and PARL osmolality was affected by diet fed to the cows (*p* < 0.001). On the other hand, saliva osmolality was not affected by diet (*p* = 0.15). Furthermore, an interaction between diet and time was observed for FRL and PARL (*p* = 0.003). Cows receiving a grain‐rich diet showed higher FRL (+24%) and PARL (+36%) osmolality compared to cows fed a forage‐based diet (*p* < 0.001). When cows were fed a grain‐rich diet at 0 h and 8 h post‐feeding, osmolality of saliva was lower than FRL and PARL (*p* < 0.05). When cows received a forage‐based diet, saliva osmolality decreased (*p* < 0.05) compared to the FRL osmolality at all sampling times (0 and 8 h post feeding), but there was no difference when compared to the PARL osmolality after 8 h post‐feeding.

**TABLE 4:** Osmolality values (mOsm/kg) of free ruminal liquid (FRL), particle associated rumen liquid (PARL) and saliva of cows fed grain‐rich and forage‐based diets.

Item	Diet				SEM^4^	*p*		
	High grain		Forage‐based					
Time^1^	0	8	0	8		Diet	Time	Interaction
FRL^2^	323^a^	333^a^	255^a^	273^a^	12.0	< 0.001	0.26	0.003
PARL^3^	299^a^	349^a^**	258^a^	217^b^*	12.0	< 0.001	0.26	0.003
Saliva	173^b^	164^b^	160^b^	216^b^*	9.65	0.15	0.37	0.053

*Note:* ^a,b^Means of sample type sharing no common superscripts at the same time point are significantly different (*p* < 0.05). *,**Means of hours after morning feeding differ from 0 h, *p* < 0.05 and *p* < 0.01, respectively.

^1^ Hours after morning feeding.

^2^ Free rumen liquid.

^3^ Particle associated rumen liquid.

^4^ The largest SEM.

## 4 Discussion

Our hypothesis in this trial stated that the ventral rumen pH will provide a good proxy for the relationship of the pH in the dorsal rumen, though differently based on the diet fed.

The current study represents a new way to interpret the vast amount of ruminal pH data generated from wireless sensor technology, too, which could provide information about the rumen and the physiological status of the host animal. Overall, the mean pH was lower in the dorsal mat than the ventral part of the rumen, regardless of the diet. Following the same trend, the total VFA concentration was higher in PARL than FRL. These results were anticipated because the rumen mat and the central rumen have a lower pH than the ventral rumen sac due to differences in both substrate fermentation rate and VFA absorption at different rumen sites (Tafaj et al. 2004; Storm and Kristensen 2010; Aschenbach et al. 2011). Moreover, mean ruminal pH decreased when cows fed a grain‐rich diet and within both rumen locations (dorsal and ventral). These results are in accordance with previous literature (Zebeli et al. 2012). The location did not affect the maximum ruminal pH whereas the minimum ruminal pH was affected. This was reflected on the amplitude ruminal pH and the effect was more prominent when cows fed a grain‐rich diet suggesting that ruminal pH takes more time to fluctuate from minimum to maximum level with an increased concentrate level in the diet. In fact, the minimum pH is valuable for examining differences that cannot be captured by the mean pH, and a minimum pH may serve as an indicator of the rumen microbial metabolism. But the minimum ruminal pH is not sufficiently described in terms of characterization of rumen health, and no threshold of the minimum pH is still available in the literature, under which rumen health may be jeopardized. Indeed, most of the studies have been concentrated on the period of time ruminal pH is below a given threshold as being more important in terms of microbial metabolism and rumen health (Dijkstra et al. 2020). Indeed, the SARA thresholds are mainly developed depending on the duration of pH measurements in the ventral rumen (Zebeli et al. 2012). For example, common SARA thresholds are also based on that measurement, such as pH < 5.8 more than 5.4 h/day (Zebeli et al. 2008) or pH < 5.6 more than 3 h/day (Plaizier et al. 2008), whereas in the reticulum the threshold suggested is pH < 6 more than 5–6 h/day (Neubauer et al. 2018). Predicting dorsal rumen pH is diagnostically valuable as it provides a site‐specific measure of the environment for fiber degrading microbes. Consequently, the ability to estimate dorsal pH offers a more physiologically relevant metric for assessing SARA risk than ventral pH, which is more variable and less directly related to the process of fiber mat maintenance.

During a 24‐h period, the *CCC* analysis revealed a high association between dorsal and ventral ruminal pH when cows fed a grain‐rich diet but low association in cows fed a forage‐based diet. The Pearson correlation coefficients were greater than *CCC* for both diets, thus indicating higher precision in predicting the pH in the dorsal part in both diets with the pH of the ventral part. However, the accuracy for predicting pH in the dorsal part with the pH in the ventral part was lower when cows fed a forage‐based diet. These observations are reflected by a positive location shift with the underestimation of the relationship of pH in the dorsal part when cows fed a forage‐based diet. This indicates that pH in the ventral part of the rumen can predict changes in pH in the dorsal part with a reproducibility of 85% only when cows fed a grain‐rich diet. The higher reproducibility could be due to the low scale shift. These data highlight the impact of VFA accumulation during high grain feeding in various rumen locations as previously reported. Moreover, the lower scale shift in cows fed a forage‐based diet showed that the SD was lower than cows fed a grain‐rich diet. This could be explained by the lower VFA production when cows were fed a forage‐based diet (Zebeli et al. 2012).

Since hindgut fermentation not only contributes to the host's total energy supply, but also negatively affects cattle health due to hindgut dysbiosis (Neubauer et al. 2020), characterizing the fecal fermentation is crucial (Gressley et al. 2011; Mao et al. 2012; Khafipour et al. 2016). Fecal pH and ruminal pH are not often correlated to each other, unless starch can bypass the rumen and cause fermentation in the hindgut (Enemark 2008). The change from a forage‐based diet to a grain‐rich diet caused an average decrease in ruminal pH at different locations (dorsal and ventral) of the rumen. Following the same trends, the fecal pH decreased by 0.22 when cows fed a grain‐rich diet. In the current study, shifting from a forage‐based to a grain‐rich diet likely increased the ruminal bypass of starch reaching the hindgut of cows, resulting in a drop of fecal pH (Li et al. 2012; Neubauer et al. 2020; Khorrami et al. 2022).

Although the total VFA production increase with feeding grain‐rich diet, only minor alterations in the VFA profile were observed. Unlike PARL and FRL, the lack of response in fecal acetate suggests that the hindgut microbial community operates independently of ruminal fluctuations. This decoupling occurs because the hindgut is fueled primary by residual fibers and substrates. In fact, the hindgut buffering capacity is most likely limited in comparison to rumen. Thus, a drop of hindgut pH as a result of VFA accumulation will not have the same beneficial effect on VFA absorption as it occurs in the rumen (Aschenbach et al. 2011; Gressley et al. 2011). In other studies, the effect of reducing acetate was likewise absent, even though increasing acetate was associated with a reduction in fecal pH (Li et al. 2012; Mao et al. 2012). Therefore, it is difficult to determine the proportionate contribution of the ruminant hindgut to the rumen acidosis complex syndrome due to the inadequate information available about it (Sanz‐Fernandez et al. 2020).

Ruminal microorganisms are prone to lysis or swelling when substantial and fast changes in osmotic pressure occur; therefore, stable osmotic pressure guarantees rumen environment stability (Youssef and Attia‐Ismail 2020). Therefore, many studies focused on the rumen osmolality changes as a result of dietary changes (Grünberg and Constable 2009; Dong et al. 2015; Chibisa et al. 2016; Osman et al. 2020; Youssef and Attia‐Ismail 2020). To our knowledge, there is no study performed to evaluate the effect of dietary changes on rumen osmolality in different locations of the rumen and its relation to saliva osmolality. In the current study, the higher ruminal osmolality in grain‐rich diet reflected the changes in the total VFA concentration that might be attributed to a higher ruminal supply of rapidly fermentable carbohydrates due to higher dietary starch content. Because of the differences within the rumen in terms of pH and fermentation, we tested the hypothesis that rumen osmolality would be different at different locations in the rumen. The osmolality of FRL was greater than PARL, which was not anticipated due to the higher concentration of VFA in the PARL. Rumen fluid osmolality can be influenced by multiple factors. Thus, the lower PARL osmolality may only be partially attributed to VFA concentration. In fact, VFA represent 30 to 40% of the total ruminal solutes (Girard et al. 2009), with the major elements contributing more than salts of VFA to rumen osmolality (Youssef and Attia‐Ismail 2020). However, the postprandial effect of high grain feeding appears to rise PARL osmolality, suggesting that the concentration of VFA could have a greater impact on rumen fluid osmolality during postprandial situation. Recently, Castillo‐Lopez et al. (2021) reported that saliva osmolality was increased by the duration of high grain feeding. The authors suggested using saliva osmolality as an indicator for acidosis occurrence and severity. However, in the current study, the correlation between saliva osmolality and rumen fluid osmolality at different locations (FRL and PARL) and under different feeding systems (grain and forage‐based diets) was weak and non‐significant. Although increasing rumen fluid osmolality results in raising osmolality of digesta down the gastrointestinal tract and blood, ruminants actively regulate rumen osmolality, which closely resembles serum osmolality (Grünberg and Constable 2009). This could explain the lack of diet effect on saliva osmolality and the absence of correlation between saliva osmolality and rumen fluid osmolality in the current study. However, this relation could be clearer with long duration of high‐grain feeding. Although we did not measure saliva composition in this trial, our group recently demonstrated that high grain feeding affects saliva physio‐chemical composition and buffering capacity (Castillo‐Lopez et al. 2021). Thus, the relationship between osmolality in different parts of the rumen and saliva osmolality as an indicator of acidosis needs further investigations, taking into account other factors such as duration of feeding, water intake, and saliva composition.

The study has also some weaknesses that need to be taken into consideration. First, the study was conducted using limited numbers of cows; whereas the number was sufficient to collect initial data, it may constrain the statistical power and limit the generalizability of the results. Moreover, a notable limitation is the lack of independent validation. Consequently, further research using independent groups is suggested to confirm the power and reproducibility of the outcomes.

Taken together, the present research suggests a relationship model of ruminal pH of the dorsal mat using measurements of the ventral ruminal pH as a proxy, but with high reproducibility only during feeding a grain‐rich diet (0.857). The data also showed that osmolality was not uniform in the rumen, and it was affected in different ways by the diet. Cows on a grain‐rich diet exhibited elevated osmolality in both the ruminal fluid (FRL and PARL) in comparison to cows fed a forage‐based diet. However, this effect needs further investigation. Salivary osmolality was not correlated with rumen osmolality, which indicates that rumen osmolality is a strongly regulated process.

## Funding

The authors have nothing to report.

## Conflicts of Interest

The authors declare no conflicts of interest.

## Supporting information


**Table S1:** Ingredients, chemical composition, and particle size distribution of the diets fed to cows during the forage and high grain feeding.
