# Supplementary material for: Characterizing Intraruminal Differences of the pH and Fermentation in Cattle
Source: Anim Sci J. 2026 Jul 26;97(1):e70226. doi: 10.1111/asj.70226 (PMC13402330; doi:10.1111/asj.70226)
Supplement: Supplementary file 1 — Table S1: Ingredients, chemical composition, and particle size distribution of the diets fed to cows during the forage and high grain feeding. [file ASJ-97-e70226-s001.docx]

**Supplementary Table 1.** Ingredients, chemical composition, and particle size distribution of the diets fed to cows during the forage and high grain feeding.

|  | Diet | |
| --- | --- | --- |
| Item | Forage based | High grain |
| Ingredients, % DM |  |  |
| Grass hay | 10.00 | 0.00 |
| Grass silage | 45.00 | 26.25 |
| Corn silage | 45.00 | 8.75 |
| Concentrate^1^ | 0.00 | 65.00 |
| TMR chemical composition |  |  |
| DM, % as fresh | 34.0 ± 1.06 | 47.1 ± 1.84 |
| Crude protein, % | 11.0 ± 0.18 | 17.8 ± 0.38 |
| Neutral detergent fiber (NDF), % | 55.5 ± 1.80 | 32.0 ± 1.90 |
| Acid detergent fiber (ADF), % | 34.2 ± 0.23 | 21.7 ± 0.85 |
| Starch, % | 17.0 ±1.75 | 28.8±1.90 |
| Ether extract, % | 1.98 ± 0.25 | 3.23 ± 0.48 |
| Non-fiber carbohydrates, % | 22.9 ± 1.62 | 39.3 ± 2.21 |
| Ash, % | 6.70 ± 0.10 | 6.76 ± 0.27 |
| Particle fraction (% retained)^2^ |  |  |
| Long | 64.56 ± 3.55 | 28.63 ± 3.14 |
| Medium | 21.30 ± 3.00 | 29.09 ± 4.63 |
| Short | 13.62 ± 0.52 | 40.14 ± 5.00 |
| Fine | 0.51 ± 0.02 | 2.13 ± 1.23 |
| pef^4^ >8 mm | 0.85 ± 0.005 | 0.58 ± 0.06 |
| peNDF^5^ >8 mm | 49.28 ± 1.53 | 18.46 ± 1.48 |

^1^The pelleted concentrate mixture contained: wheat (30.36%), triticale (18.06%), bakery by-product (23.02%), rapeseed meal (23.94%), molasses (2.99%), mineral-vitamin premix for dairy cattle (1.53%), limestone (1.0%).

^2^Particle fractions determined by Penn State Particle Separator with a 19-mm screen (long), 8-mm screen (medium), 1.18-mm screen (short), and a pan (fine) according to Kononoff et al. (2003).

^4^Physical effectiveness factor.

^5^Physically effective NDF.
